# Supplementary material for: AI-guided Cas9 engineering provides an effective strategy to enhance base editing
Source: Mol Syst Biol. 2025 Sep 15;21(11):1563–80. doi: 10.1038/s44320-025-00142-0 (PMC12583830; doi:10.1038/s44320-025-00142-0)
Supplement: Supplementary file 1 — Appendix [file 44320_2025_142_MOESM1_ESM.pdf]

Appendix for

AI-guided Cas9 engineering provides an effective strategy to enhance base editing

Table of Contents

Name	Description	page
Appendix Figure S1	Screening of candidate single-point mutants	2
Appendix Figure S2	Screening of candidate eight-points mutants	3
Appendix Figure S3	Editing efficiency comparison of AncBE4max and AncBE4max-AI-8.3 in HEK293T cells	4
Appendix Figure S4	Editing efficiency comparison of AncBE4max and AncBE4max-AI-8.3 in HeLa cells	5
Appendix Figure S5	Distribution of eight points mutations incorporated in Cas9-AI-8.3	6
Appendix Figure S6	YEE-AI-8.3 exhibits enhanced editing efficiency in HEK293T cells	6
Appendix Figure S7	Increased editing efficiency of CGBE-AI-8.3 in HEK293T cells	7
Appendix Figure S8	Associated indel ratios in ABEs	8
Appendix Figure S9	CRISPRa activities of dCas9-VPRs	8
Appendix Figure S10	Relative indel ratio and editing window in HF1-CBEs	9
Appendix Figure S11	Cleavage activity of Cas9(H840A)	9
Appendix Figure S12	Molecular Dynamics Simulation	10
Appendix Figure S13	Analysis of indel ratios generated by BEs in human embryonic stem cells and seven cancer cell lines	10
Appendix Figure S14	Zero-shot prediction of mutation effects on Cas9 datasets	11
Appendix Table S1	Top10 eight-point combination mutations	11
Appendix Table S2	Primers used for AI-guided engineered nCas9 variants construction	11
Appendix Table S3	Spacer sequences used for constructing sgRNA plasmids	12
Appendix Table S4	Primers used for cells genomic DNA amplification and targeted deep sequencing	13

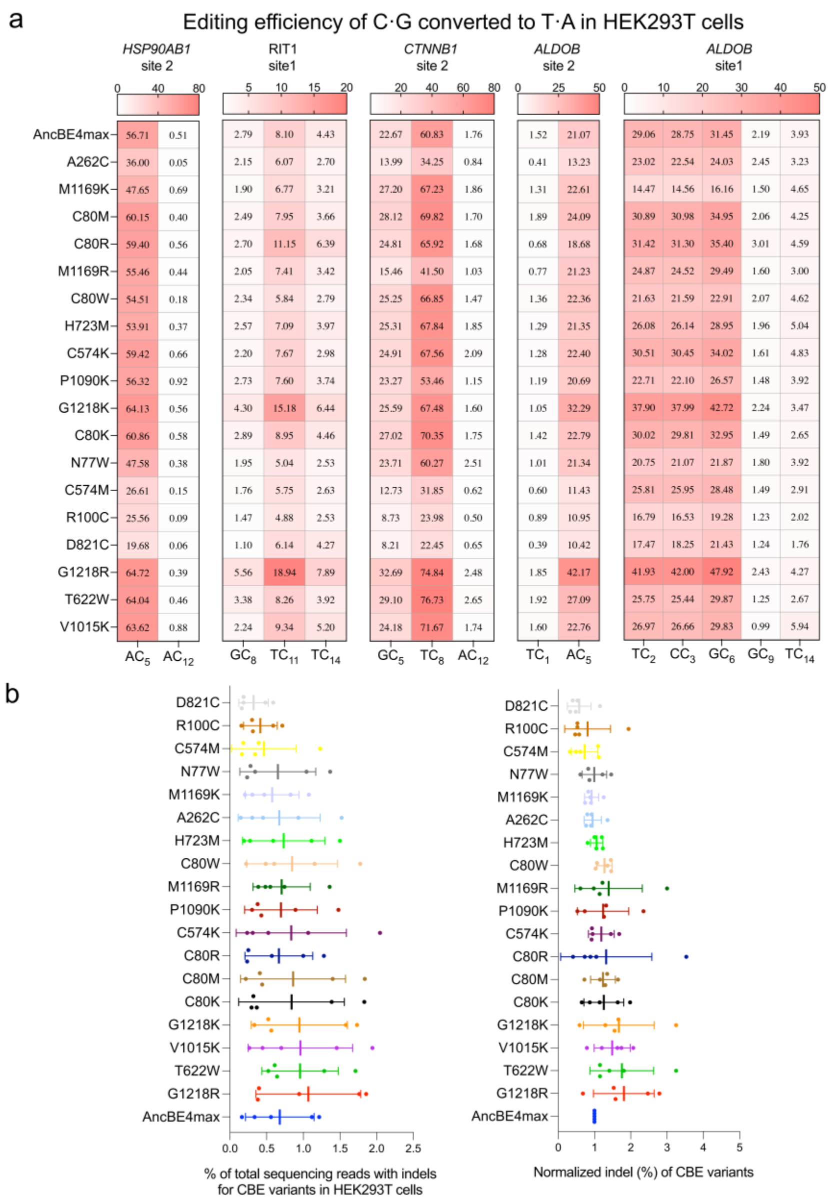

Appendix Figure S1: a. Heatmap of C-to-T conversion (%) induced by AncBE4max and 18 single-point mutants at all editing positions across 5 sites in HEK293T. Data are presented as means (n=3 biological replicates). **b.** Relative indel ratio (%) associated with (a) was shown on the left (means ± s.d., n =5). Normalized indel ratio (%) were shown on the right. The indel ratio (%) induced by AncBE4max were set as 1.

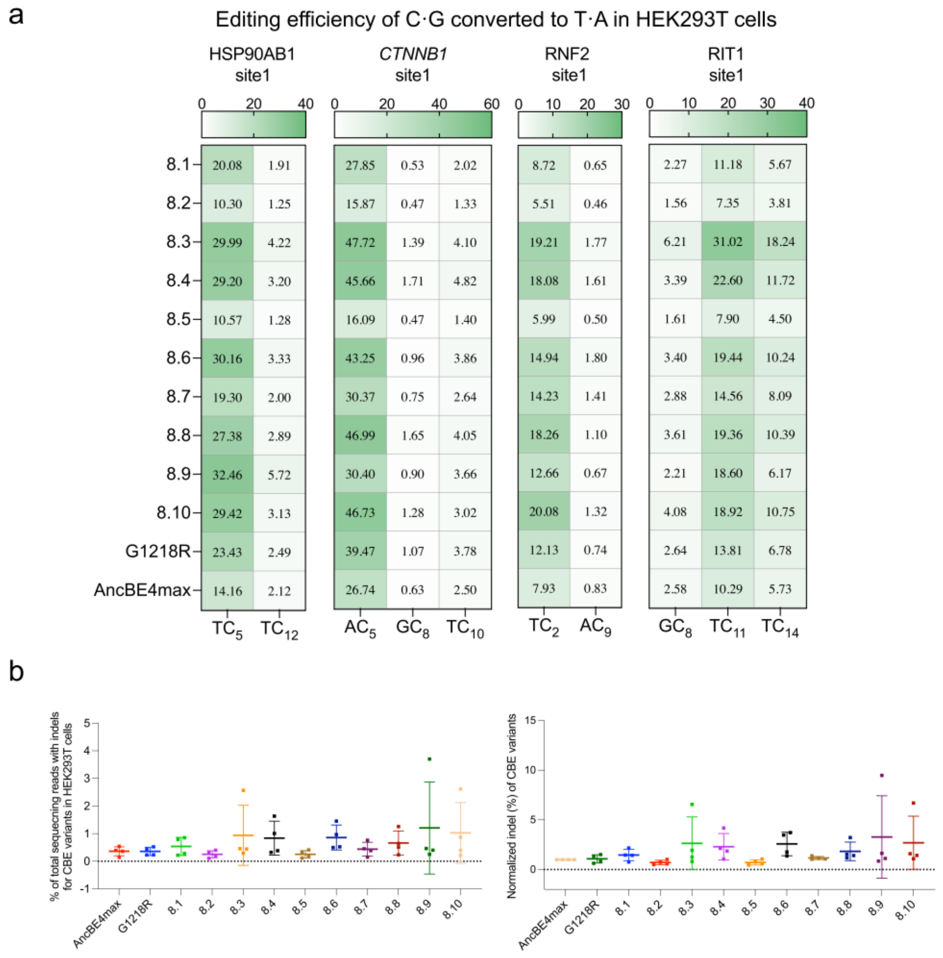

Appendix Figure S2: a. Heatmap of C-to-T conversion (%) induced by AncBE4max, G1218R and 10 eight-points mutants at all editing positions across 4 sites in HEK293T. Data are presented as means (n=3 biological replicates). **b**. Relative indel ratio (%) associated with (a) was shown on the left (means ± s.d., n = 4). Normalized indel ratio (%) were shown on the right. The indel ratio (%) induced by AncBE4max were set as 1.

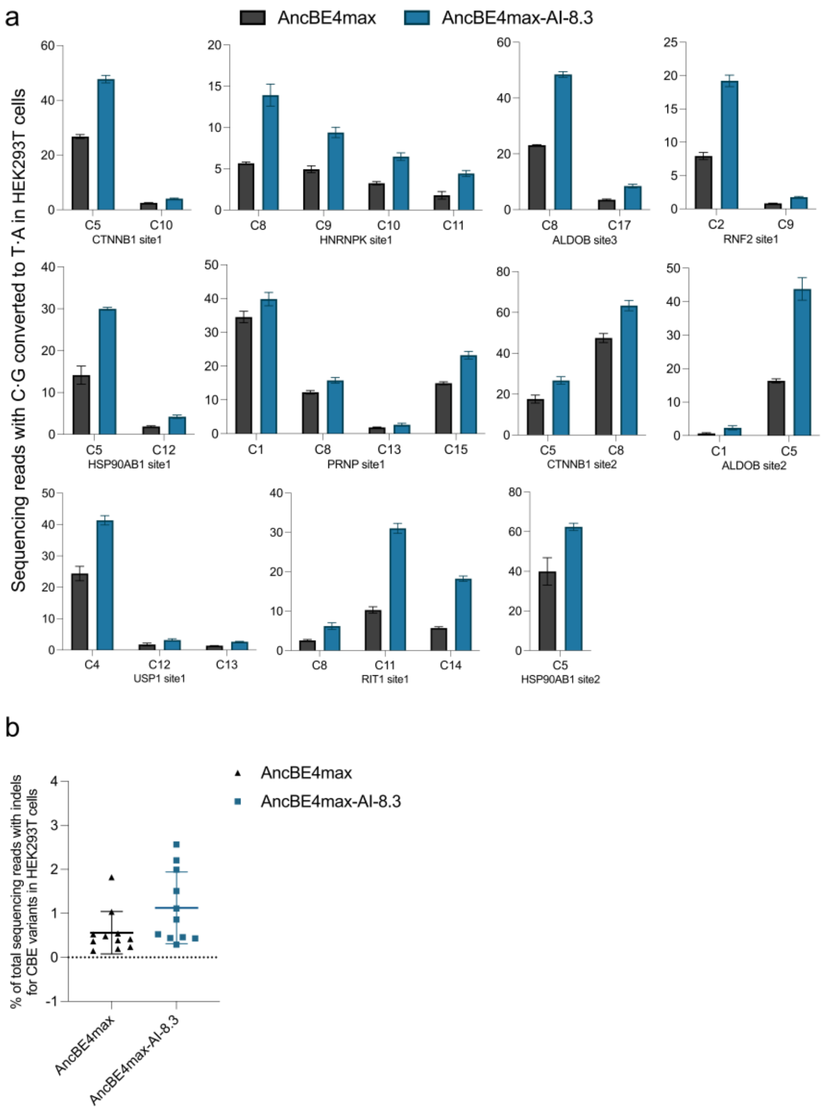

Appendix Figure S3: a. The editing efficiency of AncBE4max and AncBE4max-AI-8.3 at all editing positions across 11 sites in HEK293T cells. **b**. Relative indel ratio (%) of AncBE4max and AncBE4max-AI-8.3 associated with (a) (means ± s.d., n = 11) (p>0.05, Two-tailed, Unpaired t-test).

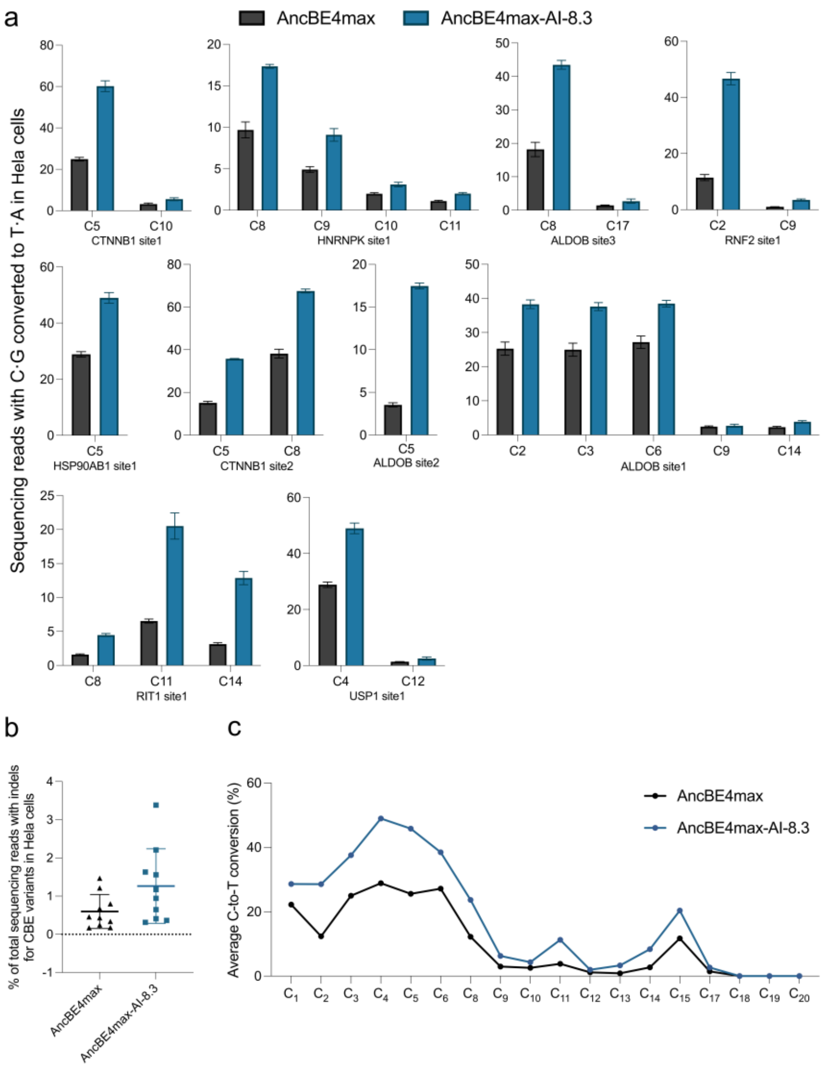

Appendix Figure S4: a. The editing efficiency of AncBE4max and AncBE4max-AI-8.3 at all editing positions across 10 sites in HeLa cells. **b**. Relative indel ratio (%) of AncBE4max and AncBE4max-AI-8.3 associated with (a) (means ± s.d., n =10) ($p>0.05$, Two-tailed, Unpaired t-test). **c**. Summary of average C-to-T conversion (%) for AncBE4max and AncBE4max-AI-8.3 at protospacer positions (1-20) in 11 endogenous sites of HeLa cells (n=11).

a

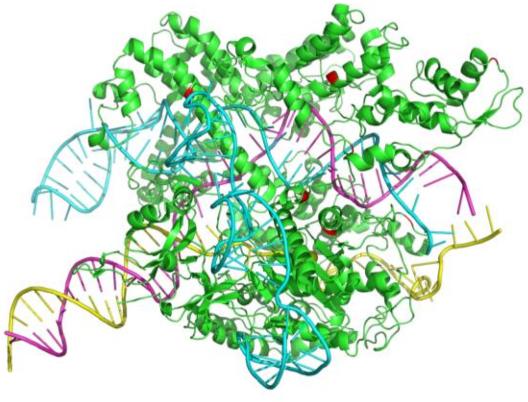

SpCas9(AI-8.3)

b

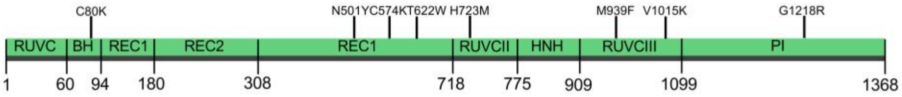

Appendix Figure S5: **a**. The resolved protein-sgRNA-DNA structure (PDB:8G1I). Mutations contained in Cas9-AI-8.3 are highlighted in red. **b**. Linear domain arrangement of Cas9, with mutated sites marked by black solid lines.

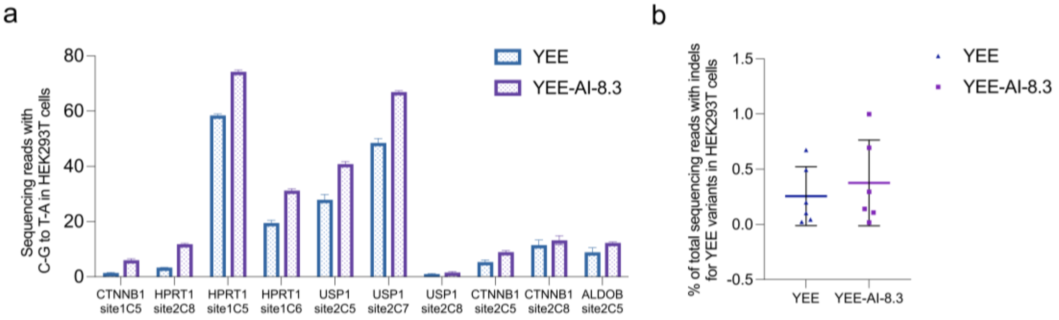

Appendix Figure S6: **a**. Sequencing reads with C-to-T of YEE-BE4max and YEE-AI-8.3 at all editing positions across 6 sites in HEK293T cells (mean±s.d., n=3 biological replicates). **b**. Relative indel ratio (%) of YEE-BE4max and YEE-AI-8.3 associated with (a) (means ± s.d., n =6) (p>0.05, Two-tailed, Unpaired t-test).

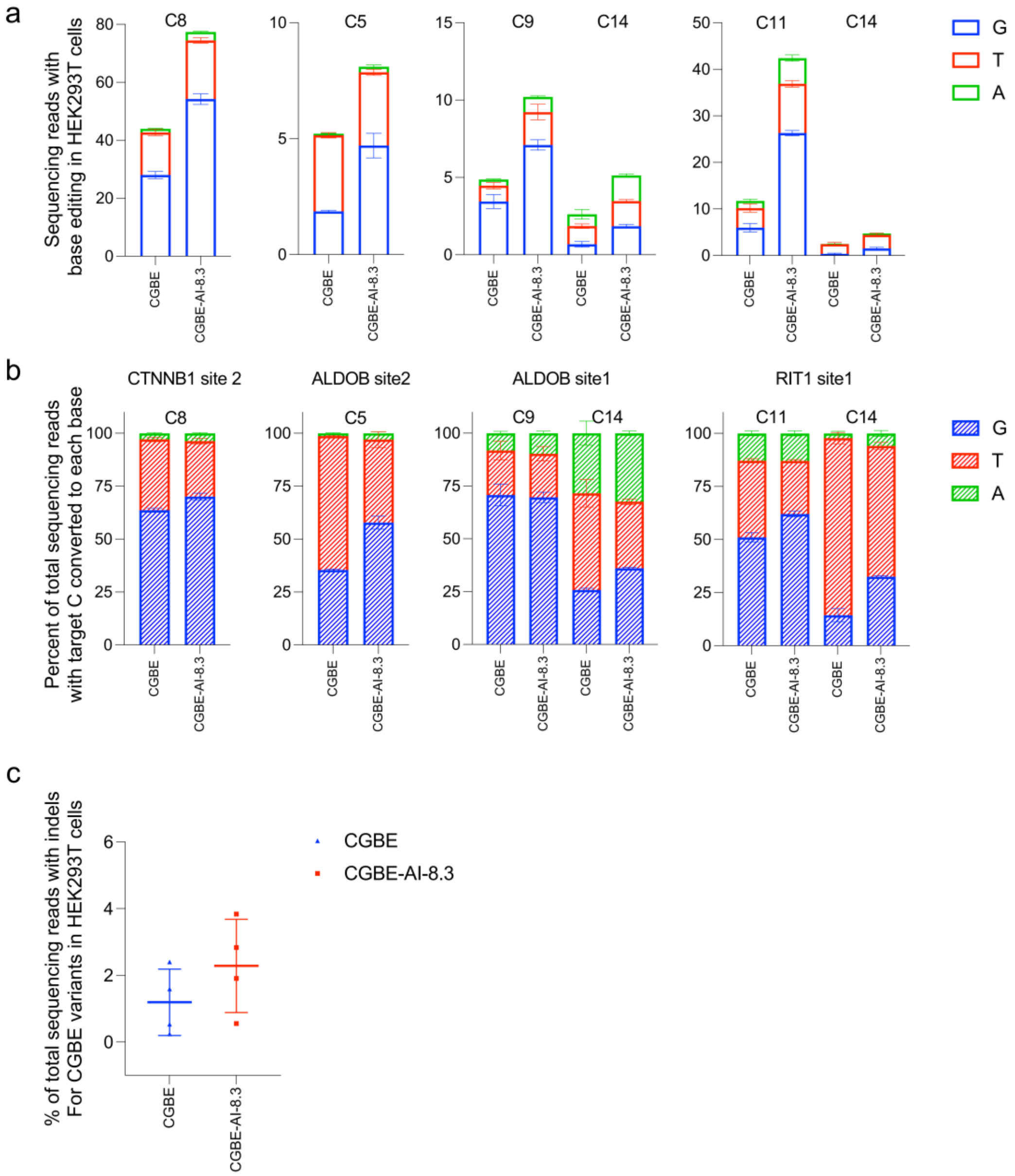

Appendix Figure S7: a. The percent of total sequencing reads with target C convened to each base at 4 sites in HEK293T cells were shown. **b**. The distribution of C converted to each base among edited DNA sequencing reads at the same sites in (a) were shown. **c**. Relative indel ratio (%) of CGBE and CGBE-AI-8.3 associated with (a) (means ± s.d., n = 4) (p>0.05, Two-tailed, Unpaired t-test).

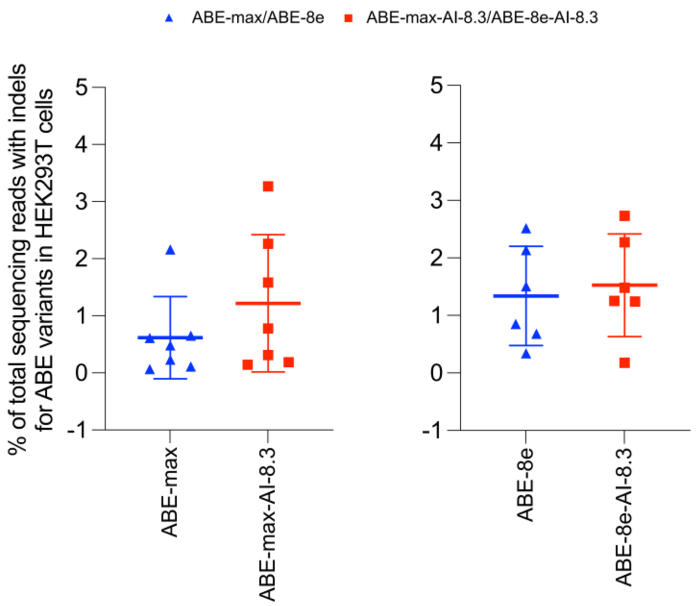

Appendix Figure S8: Relative indel ratio (%) of ABE-max, ABE-max-AI-8.3, ABE-8e and ABE-8e-AI-8.3 associated with Figure 3F and Figure 3H in HEK293T cells (means ± s.d., n =9 (ABE-max) or 6 (ABE-8e)) (p>0.05, Two-tailed, Unpaired t-test).

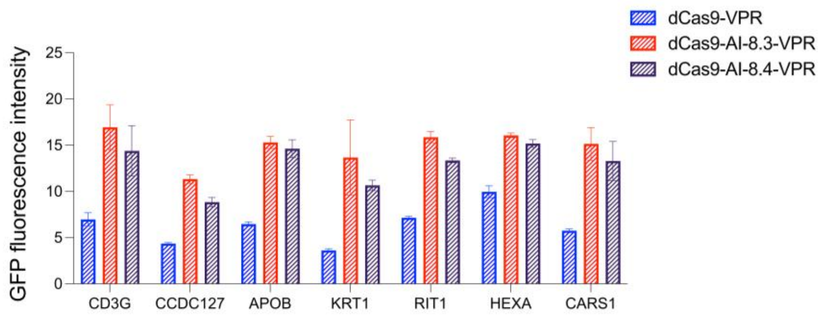

Appendix Figure S9: CRISPRa activity was assessed using normalized reporter expression, defined as the ratio of cells displaying equivalent high EGFP fluorescence to the total mCherry-positive cell population (means ± s.d., n = 3 biological replicates). Data shown were derived from FlowJo (v10).

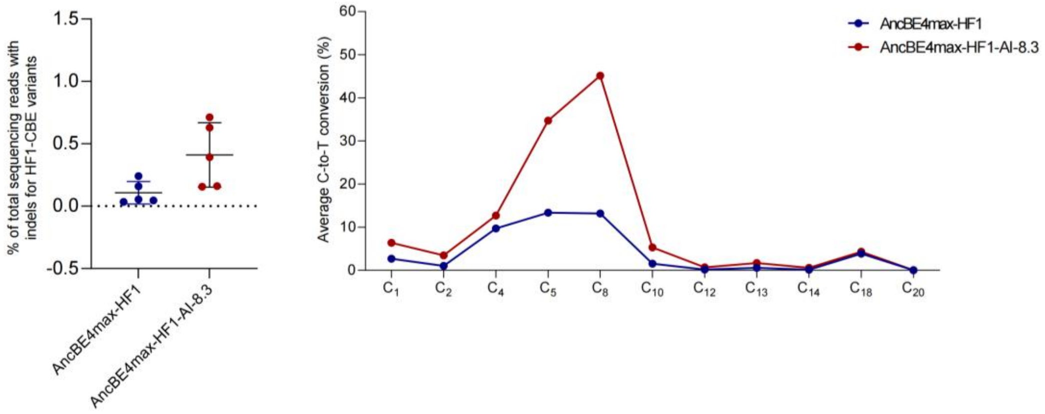

Appendix Figure S10: Relative indel ratio (%) of AncBE4max-HF1 and AncBE4max-HF1-AI-8.3 (means ± s.d., n=5) (p=0.0382, Two-tailed, Unpaired t-test). The right panel indicated the summary of average C-to-T conversion (%) for AncBE4max-HF1 and AncBE4max-HF1-AI-8.3 at protospacer positions in 5 endogenous sites of HEK393T cells.

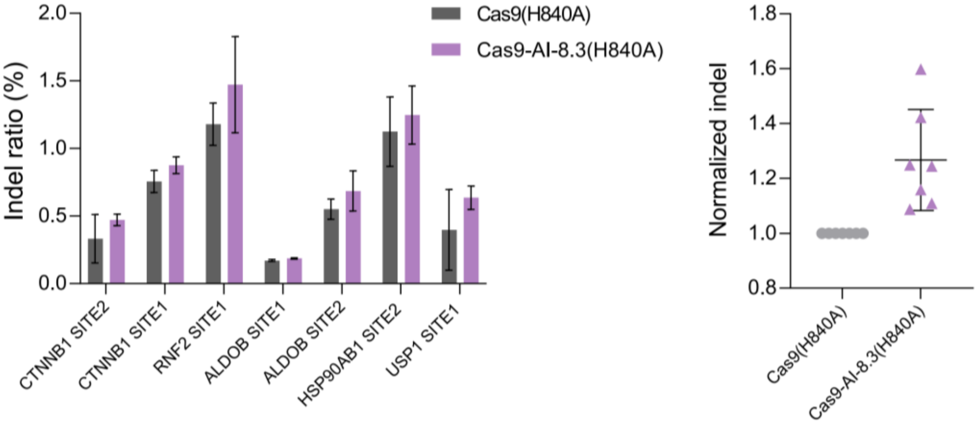

Appendix Figure S11: The indel ratio (%) of Cas9 (H840A) and Cas9-AI-8.3 (H840A) at seven genomic sites (mean±s.d., n=3 biological replicates). The right panel indicated the summary of Indel ratio. The indel rates (%) induced by Cas9 (H840A) were set as 1.

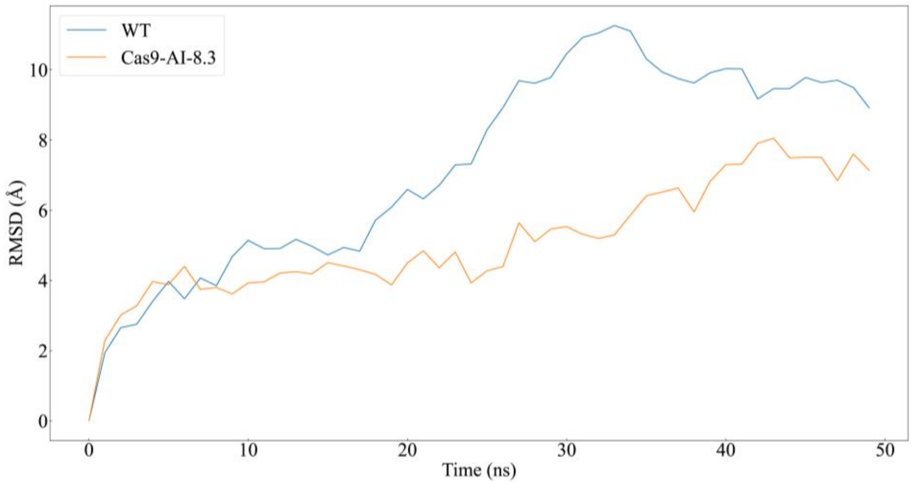

Appendix Figure S12: RMSD of WT Cas9 (blue) and Cas9-AI-8.3 (orange) during 50 ns MD simulations.

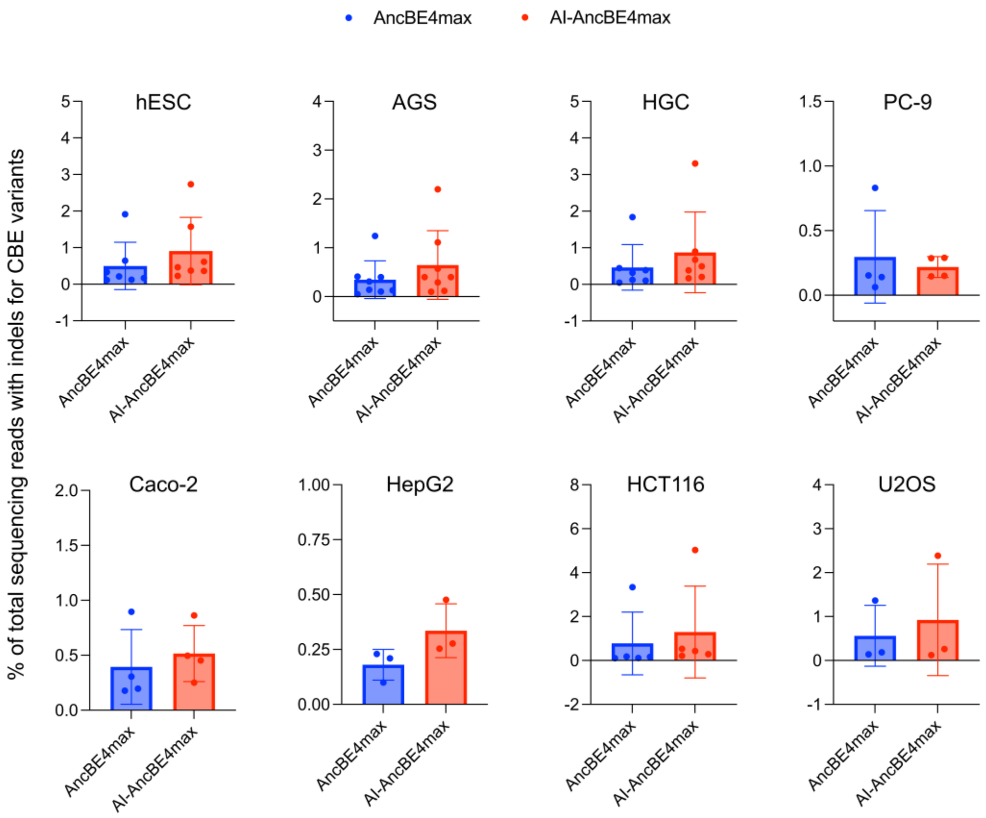

Appendix Figure S13: Total sequencing reads with indel induced by AncBE4max and AI-AncBE4max at all sites in Figure 5B and Figure 5D (means ± s.d., n = 7, 8, 7, 4, 4, 3, 5 and 3, respectively) ($p>0.05$, Two-tailed, Unpaired t-test).

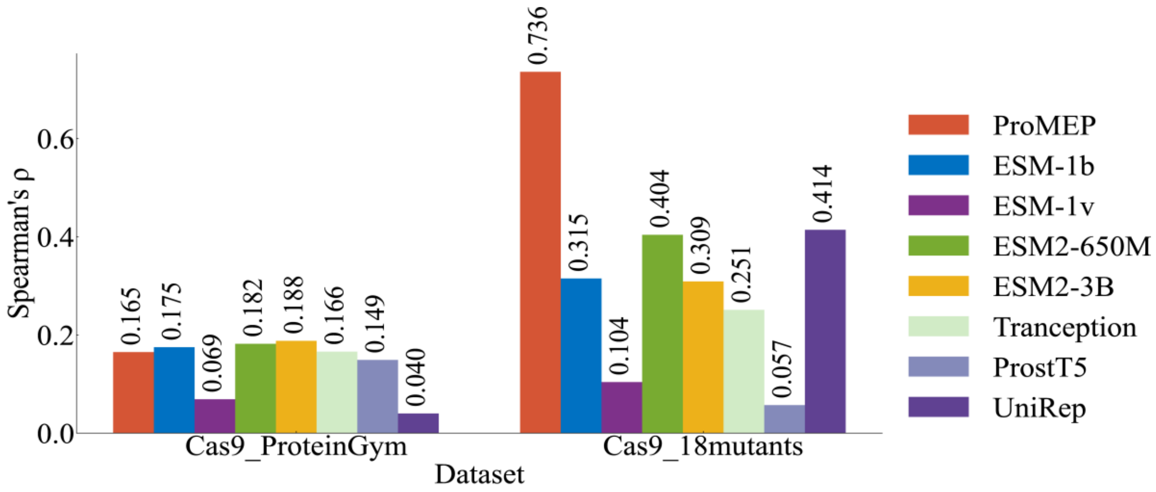

Appendix Figure S14: Zero-shot prediction of mutation effects on Cas9 datasets.

Appendix Table S1

AncBE4max-AI-8.1	C80K:G1218R:T622W:C574K:V1015K:N501Y:M939F:H723V
AncBE4max-AI-8.2	C80K:G1218R:T622W:C574K:V1015K:N501Y:M939F:M1169K
AncBE4max-AI-8.3	C80K:G1218R:T622W:C574K:V1015K:N501Y:M939F:H723I
AncBE4max-AI-8.4	C80K:G1218R:T622W:C574K:V1015K:N501Y:M939F:H723L
AncBE4max-AI-8.5	C80K:G1218R:T622W:C574K:V1015K:N501Y:M939F:V824T
AncBE4max-AI-8.6	C80K:G1218R:T622W:C574K:V1015K:N501Y:M939F:P1090K
AncBE4max-AI-8.7	C80K:G1218R:T622W:C574K:V1015K:N501Y:M939F:P1137T
AncBE4max-AI-8.8	C80K:G1218R:T622W:C574K:V1015K:N501Y:M939F:N980D
AncBE4max-AI-8.9	C80K:G1218R:T622W:C574K:V1015K:N501Y:M939F:G485E
AncBE4max-AI-8.10	C80K:G1218R:T622W:C574K:V1015K:N501Y:M939F:P28I

Appendix Table S2

NO.	Forward primer	Reverse primer
A262C	TGTAAACTGCAGCTGAGCAAGGACACCTACGACGACGACC	CTCAGCTGCAGTTTACAATCCTCGGCCAGGTCGAAGTTGC
M1169K	AAGGAAAGAAGCAGCTTCGAGAAGAATCCCATCGACTTTCTG	AGCTGCTTCTTTCCTTGATGGTGATCCCCAGCAGCTCTTTC
C80M	ATGTATCTGCAAGAGATCTTCAGCAACGAGATGGCCAAGGTG	TCTCTTGCAGATACATGATCCGGTTCTTCCGTCTGGTGTATC
C80R	AGATATCTGCAAGAGATCTTCAGCAACGAGATGGCCAAG	ATCTCTTGCAGATATCTGATCCGGTTCTTCCGTCTGGTG
M1169R	AGAGAAAGAAGCAGCTTCGAGAAGAATCCCATCGACTTTC	AAGCTGCTTCTTTCTCTGATGGTGATCCCCAGCAGCTCT
C80W	TGGTATCTGCAAGAGATCTTCAGCAACGAGATGGCCAAGGTG	TCTCTTGCAGATACCAGATCCGGTTCTTCCGTCTGGTGTATC
H723M	ATGATTGCCAATCTGGCCGGCAGCCCCGCCATTAAGAAGGG	CCAGATTGGCAATCATCTCGTGCAGGCTATCGCCCTGGCCG

C574K	AAGTTCGACTCCGTGGAAATCTCCGGCGTGGAAGATCGGTTC	CCACGGAGTCGAACTTCTCGATTTTCTTGAAGTAGTCCTC
P1090K	AAGCAAGTGAATATCGTGAAAAAGACCGAGGTGCAGACAG	CGATATTCACTTGCTTCATGCTCAGCACTTTCCGCACGGTG
G1218K	AAGGAACTGCAGAAGGGAAACGAACTGGCCCTGCCCTCCA	CCCTTCTGCAGTTCCTTGGCAGAGGCCAGCATTCTCTTCC
C80K	AAGTATCTGCAAGAGATCTTCAGCAACGAGATGGCCAAGGTG	TCTCTTGCAGATACTTGATCCGGTTCTTCCGTCTGGTGTATC
N77W	TGGCGGATCTGCTATCTGCAAGAGATCTTCAGCAACGAGATG	GATAGCAGATCCGCCACTTCCGTCTGGTGTATCTTCTTCTG
C574M	ATGTTCGACTCCGTGGAAATCTCCGGCGTGGAAGATCGG	TCCACGGAGTCGAACATCTCGATTTTCTTGAAGTAGTCCT
R100C	TGTCTGGAAGAGTCCTTCCTGGTGGAAGAGGATAAGAAGC	AAGGACTCTTCCAGACAGTGGAAGAAGCTGTCGTCCACC
D821C	TGTATGTACGTGGACCAGGAACTGGACATCAACCGGCTGT	TGGTCCACGTACATACACCGCCCATTCTGCAGGTAGTAC
G1218R	AGGGAACTGCAGAAGGGAAACGAACTGGCCCTGCCCTCC	TTTCCCTTCTGCAGTTCCCTGGCAGAGGCCAGCATTCTC
T622W	TGGCTGACACTGTTTGAGGACAGAGAGATGATCGAGGAAC	CAAACAGTGTCAGCCACAGCACGATATCTTCCAGAATGTCC
V1015K	AAGTACGACGTGCGGAAGATGATCGCCAAGAGCGAGCAGG	TCCGCACGTCGTACTTCTTGTAGTCGCCGTACACGAACTC
N501Y	TATCTGCCCAACGAGAAGGTGCTGCCCAAGCACAGCCTGC	TCTCGTTGGGCAGATACTTATCGAAGTTGGTCATCCGCTC
M939F	TTCAACACTAAGTACGACGAGAATGACAAGCTGATCCGG	TCGTCGTACTTAGTGTTGAACCGGGAGTCCAGGATCTGTG
H723V	GTTATTGCCAATCTGGCCGGCAGCCCCGCCATTAAGAAGG	CCAGATTGGCAATAACCTCGTGCAGGCTATCGCCCTGGCC
H723I	ATCATTGCCAATCTGGCCGGCAGCCCCGCCATTAAGAAGG	CCGGCCAGATTGGCAATGATCTCGTGCAGGCTATCGCCCT
H723L	TTGATTGCCAATCTGGCCGGCAGCCCCGCCATTAAGAAGG	CCGGCCAGATTGGCAATCAACTCGTGCAGGCTATCGCCCT
V824T	ACGGACCAGGAACTGGACATCAACCGGCTGTCCGACTACG	ATGTCCAGTTCCTGGTCCGTGTACATATCCCGCCCATTCT
P1137T	ACCACCGTGGCCTATTCTGTGCTGGTGGTGGCCAAAGTGG	ACAGAATAGGCCACGGTGGTGCTGTCGAAGCCGCCGTACT
N980D	GACTACCACCACGCCCACGACGCCTACCTAAACGCCGTCG	TCGTGGGCGTGGTGGTAGTCGTTGATCTCGCGCACTTTGT
G485E	GAGGCTTCCGCCCAGAGCTTCATCGAGCGGATGACCAACT	AAGCTCTGGGCGGAAGCCTCCTTGTCCACCACTTCCTCGA
P28I	ATCAGCAAGAAATTCAAGGTGCTGGGCAACACCGACCGGC	ACCTTGAATTTCTTGCTGATCACCTTGTACTCGTCGGTGA

Appendix Table S3

NO.	Forward primer	Reverse primer
HSP90AB1 site2	ACCGTTGACTTTAAACTTGTTGGC	AAACGCCAACAAGTTTAAAGTCAA
RIT1 site1	ACCGTTATAAGCATCTTCTACAGG	AAACCCTGTAGAAGATGCTTATAA
CTNNB1 site2	ACCGGCAGCATCAAACTGTGTAGA	AAACTCTACACAGTTTGATGCTGC
ALDOB site1	ACCGTCCTGCAGCTGTTCCTGGTA	AAACTACCAGGAACAGCTGCAGGA
ALDOB site2	ACCGCCTACTAGAAGCACTGGAGC	AAACGCTCCAGTGCTTCTAGTAGG
HSP90AB1 site1	ACCGCAGTCTGAACTCACTGTCTA	AAACTAGACAGTGAGTTCAGACTG
CTNNB1 site1	ACCGGAAACAGCTCGTTGTACCGC	AAACGCGGTACAACGAGCTGTTTC
RNF2 site1	ACCGTCTAGATACATAAAGACTTC	AAACGAAGTCTTTATGTATCTAGA
HNRNPK site1	ACCGCATTGAACCCCACTGAAGTA	AAACTACTTCAGTGGGGTTCAATG
ALDOB site3	ACCGCAAAGGACAGTATGTTCACA	AAACTGTGAACATACTGTCCTTTG
PRNP site1	ACCGCTGGTTGCTGTACTCATCCA	AAACTGGATGAGTACAGCAACCAG
USP1 site1	ACCGAAACAAATTTACCAAGGGAA	AAACTTCCCTTGGTAAATTTGTTT
HPRT1 site2	ACCGAATAAATCAAGGTCATAACC	AAACGGTTATGACCTTGATTTATT
HPRT1 site1	ACCGCTGTCCATAATTAGTCCATG	AAACCATGGACTAATTATGGACAG

USP1 site2	ACCGTTTACTCCAGATTTAAAACC	AAACGGTTTTAAATCTGGAGTAAA
CTLA4 site1	ACCGGCTCACCAATTACATAAATC	AAACGATTTATGTAATTGGTGAGC
CTLA4 site2	ACCGCACTCACCTTTGCAGAAGAC	AAACGTCTTCTGCAAAGGTGAGTG
HSD17B4 site1	ACCGTATTCATGGGCTCACCGCTG	AAACCAGCGGTGAGCCCATGAATA
USP1 site3	ACCGGAAAGTCCATTACTTTCACT	AAACAGTGAAAGTAATGGACTTTC
GCLC site1	ACCGGATCTACGAACAGCTGTTGC	AAACGCAACAGCTGTTCGTAGATC
Site A	ACCGCCTGCCCTGCATTTTATCAA	AAACTTGATAAAATGCAGGGCAGG
Site B	ACCGGAACACAAAGCATAGACTGC	AAACGCAGTCTATGCTTTGTGTTC
Site C	ACCGAGGGCAAACTCGCTGAAATC	AAACGATTTCAGCGAGTTTGCCCT

Appendix Table S4

NO.	Forward primer	Reverse primer
HSP90AB1 site2	GGAGGCTCTTCAGGTATTGC	GAAGACTCCCAAGCATACTG
RIT1 site1	GGATACAATGTACTACTCTG	CTGGGATTTAATACCTGTCC
CTNNB1 site2	TGGACAGTATGCAATGACTC	TGGTCCTCGTCATTTAGCAG
ALDOB site1	TGCTAAAGCCCAACATGGTG	TGCCTGTGAGATAACAGAGG
ALDOB site2	TCTAGGCTAACTGCCAGGCG	ATGTGTTGTATTTCCAGCAG
HSP90AB1 site1	TCCTTCTGAATTTTCAGGCC	TCTCCATGGTGCACTTCCTC
CTNNB1 site1	TGCAGTTATGGTCCATCAGC	TACCCAAGCATTTTCACCAG
RNF2 site1	TCAATAATTGAGACATTGCC	CTGTATCAAGGTTCATCTGG
HNRNPK site1	TCTACTTTCAGGGCCTGCAG	GCAGGGTCAACAGTGAGTTG
ALDOB site3	TTCCCATGAGAGGCAGACAG	TAGAAGCACTGGAGCTAGGC
PRNP site1	CATGAGCAGGCCCATCATAC	TCATCTTAACGTCGGTCTCG
USP1 site1	GTTCTTTAAGAACAGTGTGG	ATCCCAAGGAGTGTCAAGAG
HPRT1 site2	CCAATCAAATGTTTGTATCC	AGAACAGCTGCTGATGTTTG
HPRT1 site1	CAGATTAGTGATGATGAACC	AGTTGGCCTAGTTTATGTTC
USP1 site2	GCTCTATAGAAAGCATGATG	TTCCAGGCAAGTAAATTTCC
CTLA4 site1	GTGAACCTCACTATCCAAGG	AAGGCTACCATGTGGTACAG
CTLA4 site2	CCGCTCCCATAAAGCCATGG	AGCCAGATTGGAGTTTTACC
HSD17B4 site1	CAAGTCACTGACCCTCGTCC	TATGCCGGCCCGAAAAGAGC
USP1 site3	GGTGATTACAACTTTCCTCT	GATGCTCTATATTCAGAAGC
GCLC site1	CCCGATATGACTCAATAGAC	TAGGAACAAGTAGCCTCATG
Site A	CTGGAAGATCTTGAACCCTC	GTACTGTTGTTCAAAGTTCC
Site B	GGAATGAATGGATTCCTTGG	GTGAGAAGCCAGTGGAATAC
Site C	CCCTTAGTCTCAGCTCAGCC	TCCTGTGGAAGTCACCAACC
