# Supplementary figures and images for: AI-guided Cas9 engineering provides an effective strategy to enhance base editing
Source: Mol Syst Biol. 2025 Sep 15;21(11):1563–80. doi: 10.1038/s44320-025-00142-0 (PMC12583830; doi:10.1038/s44320-025-00142-0)

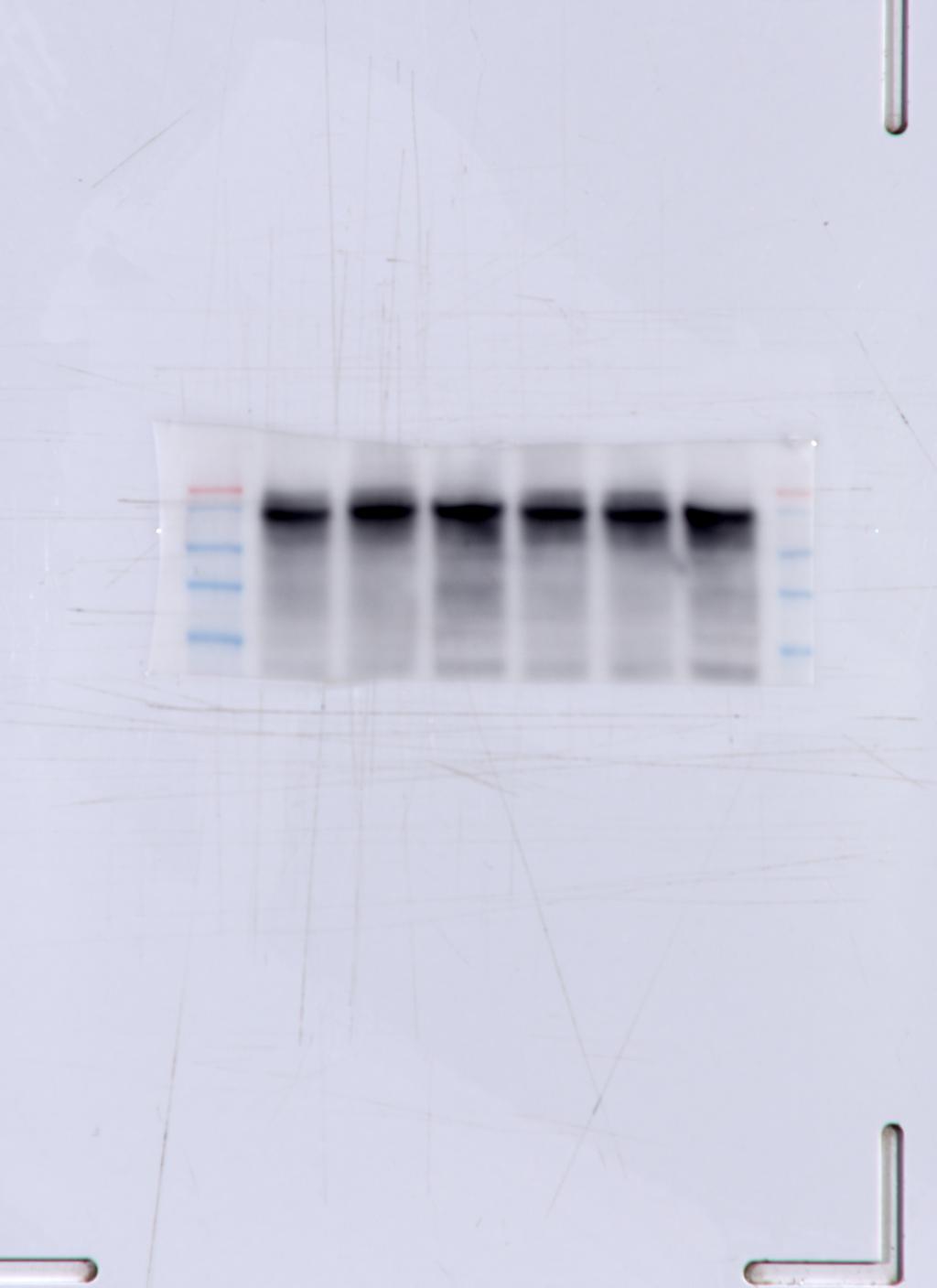

Supplement: Supplementary file 7 — Source data Fig. 4 [file 44320_2025_142_MOESM7_ESM.zip › Figure 4/4E/cas9 _20.31.35_Ch+Marker.jpg]

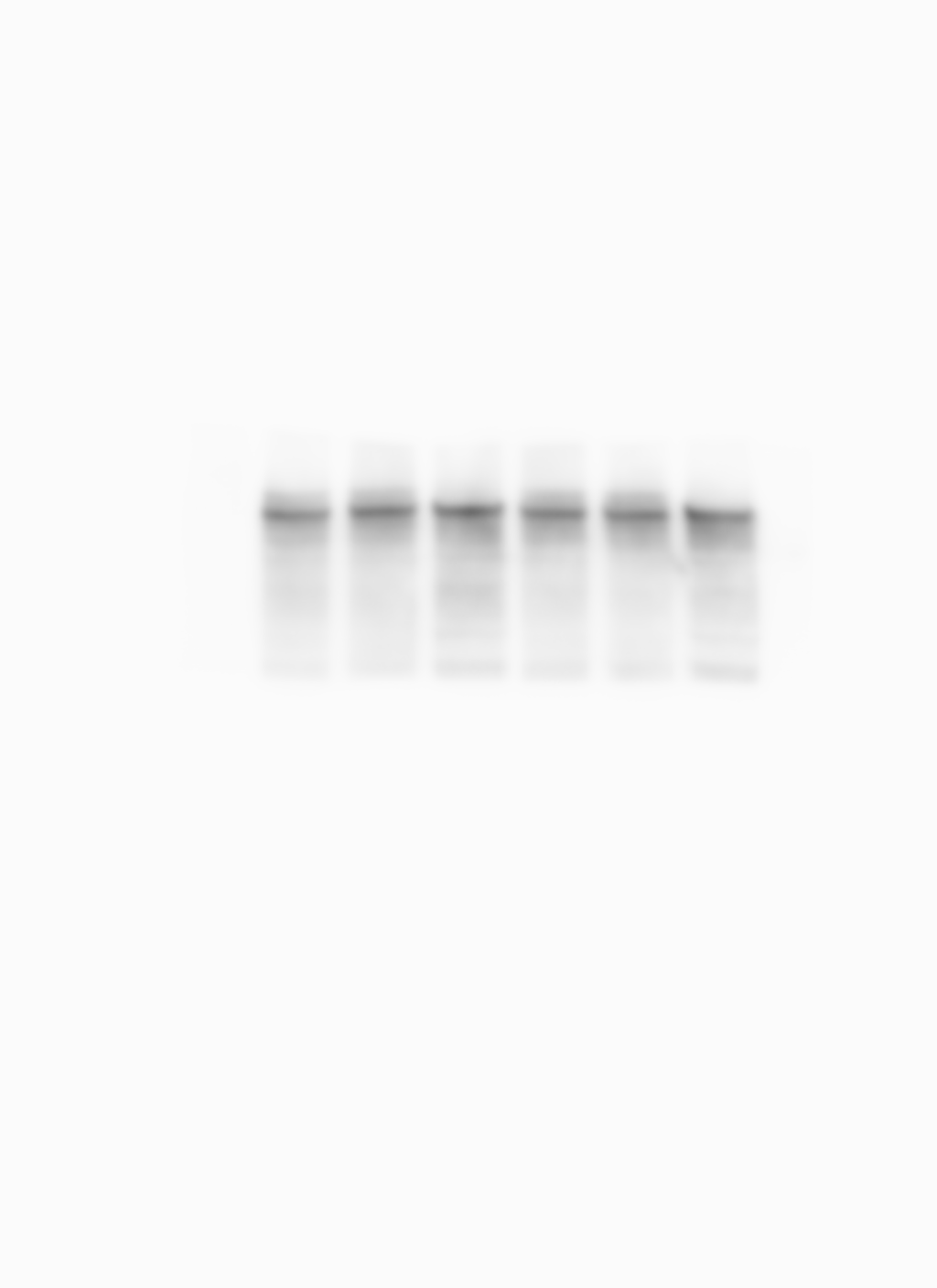

Supplement: Supplementary file 7 — Source data Fig. 4 [file 44320_2025_142_MOESM7_ESM.zip › Figure 4/4E/cas9 _20.31.35_Ch.tif]

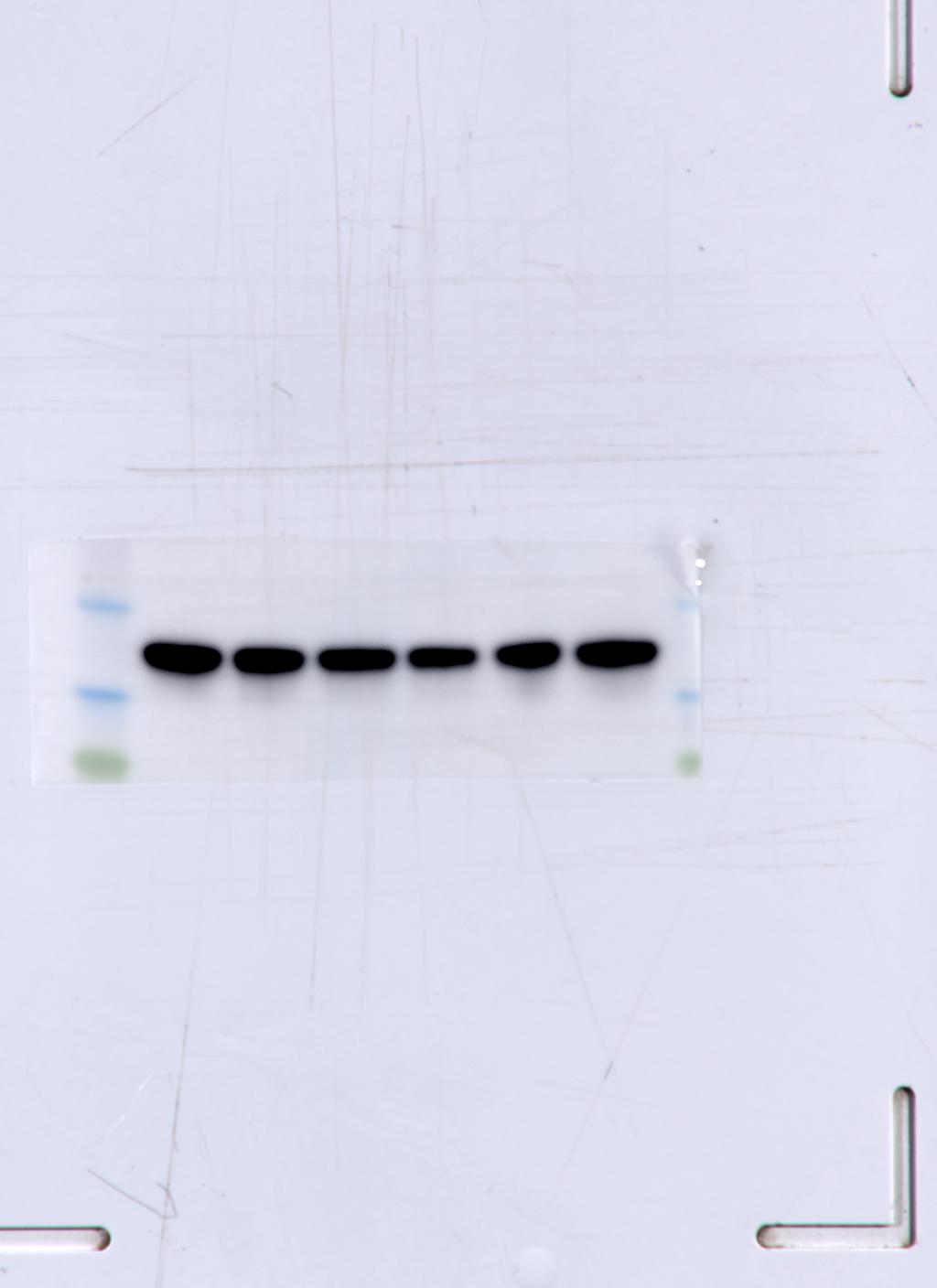

Supplement: Supplementary file 7 — Source data Fig. 4 [file 44320_2025_142_MOESM7_ESM.zip › Figure 4/4E/GAPDH_20.25.23_Ch+Marker.jpg]

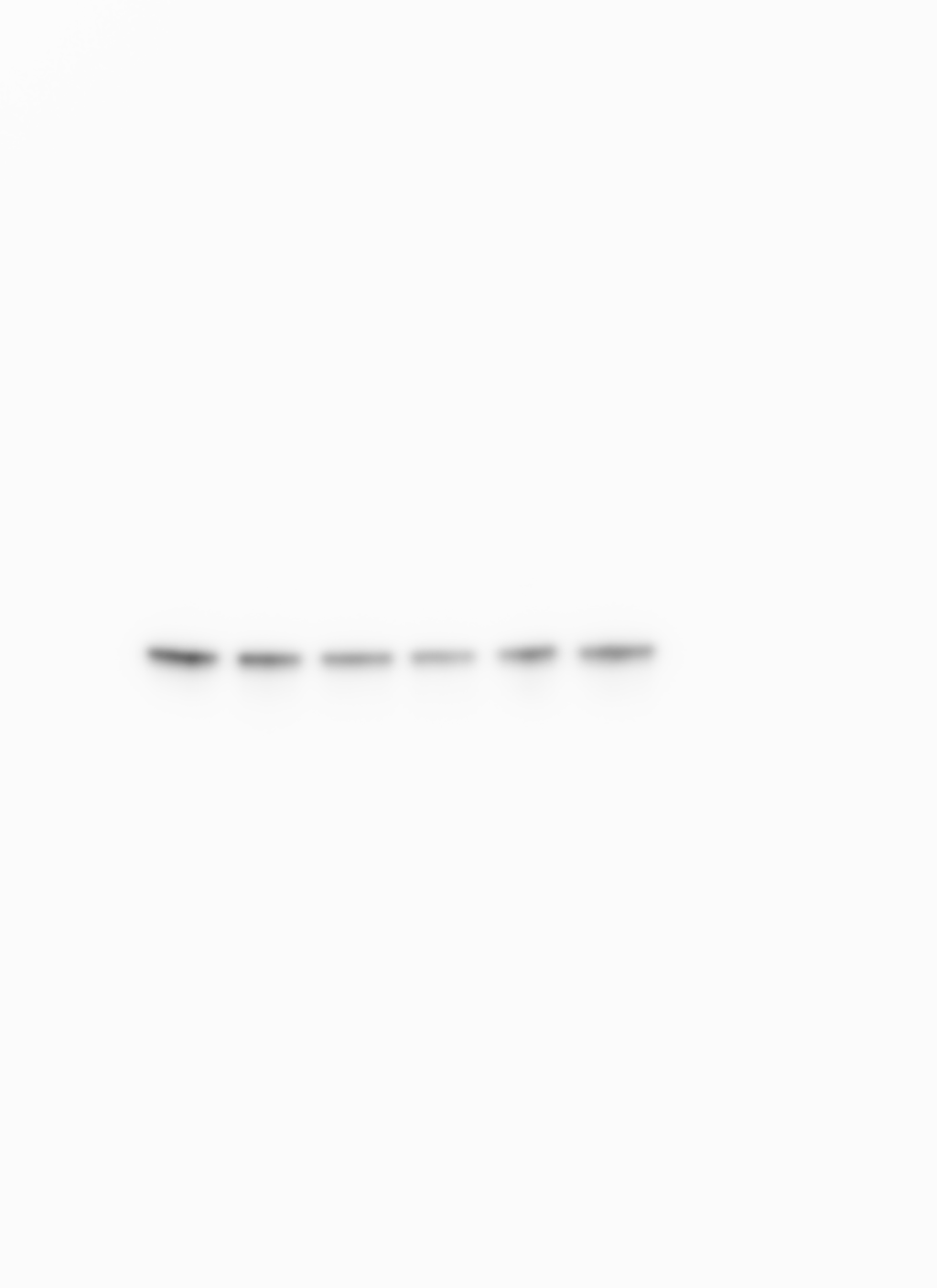

Supplement: Supplementary file 7 — Source data Fig. 4 [file 44320_2025_142_MOESM7_ESM.zip › Figure 4/4E/GAPDH_20.25.23_Ch.tif]

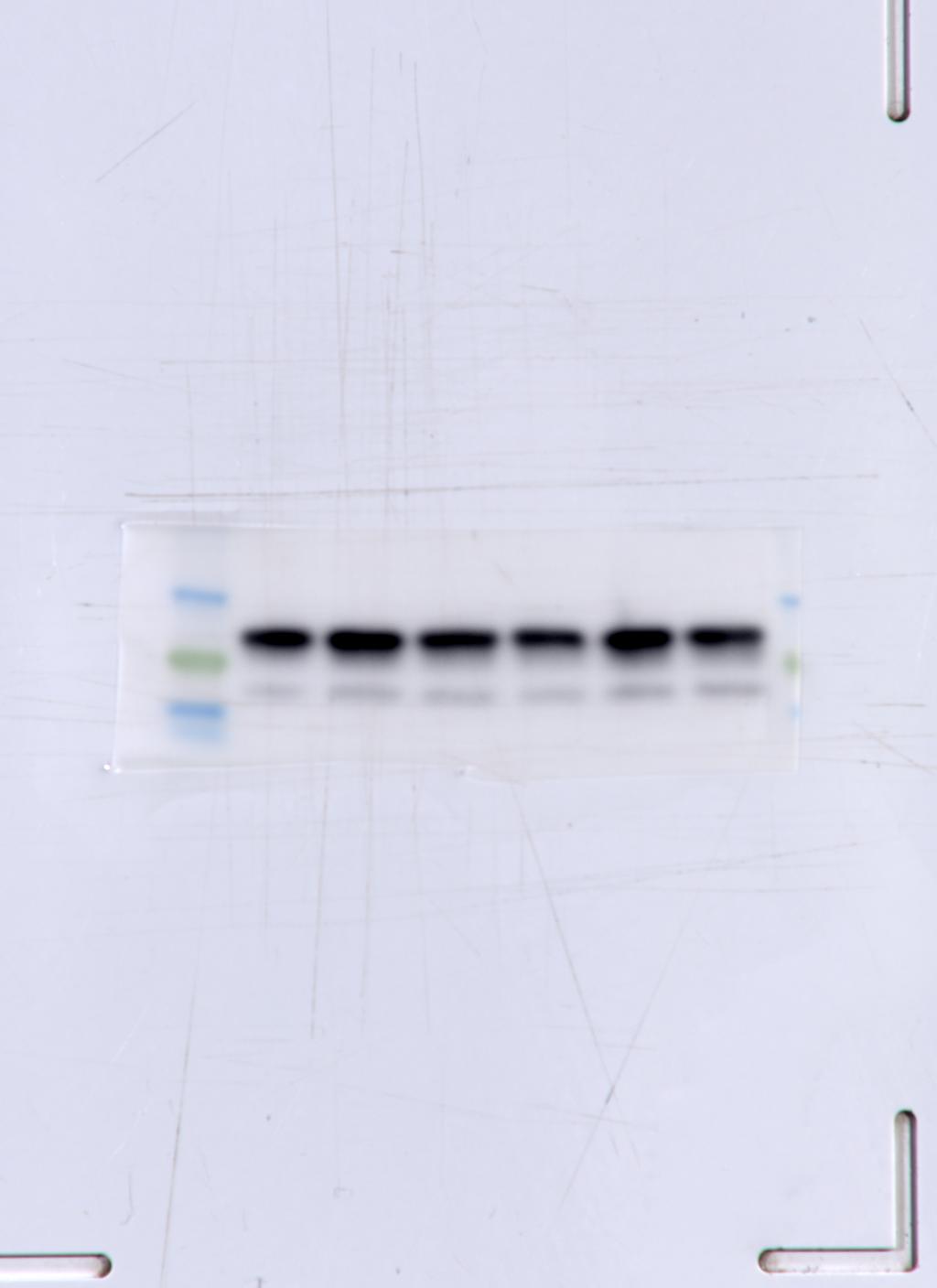

Supplement: Supplementary file 7 — Source data Fig. 4 [file 44320_2025_142_MOESM7_ESM.zip › Figure 4/4E/GFP_20.28.02_Ch+Marker.jpg]

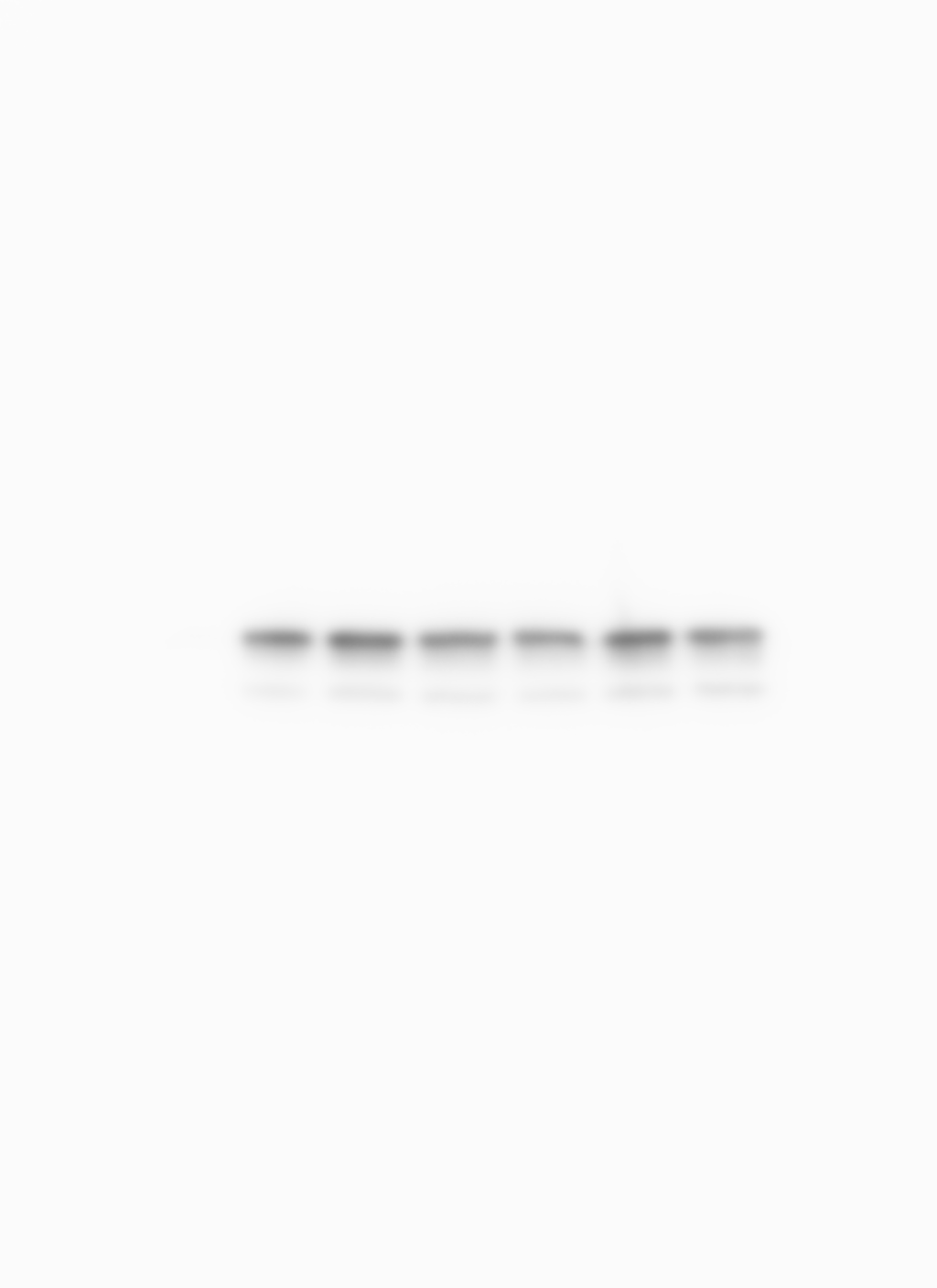

Supplement: Supplementary file 7 — Source data Fig. 4 [file 44320_2025_142_MOESM7_ESM.zip › Figure 4/4E/GFP_20.28.02_Ch.tif]
