# Supplementary material for: AI-guided Cas9 engineering provides an effective strategy to enhance base editing
Source: Mol Syst Biol. 2025 Sep 15;21(11):1563–80. doi: 10.1038/s44320-025-00142-0 (PMC12583830; doi:10.1038/s44320-025-00142-0)
Supplement: Supplementary file 9 — Expanded View Figures [file 44320_2025_142_MOESM9_ESM.pdf]

Expanded View Figures

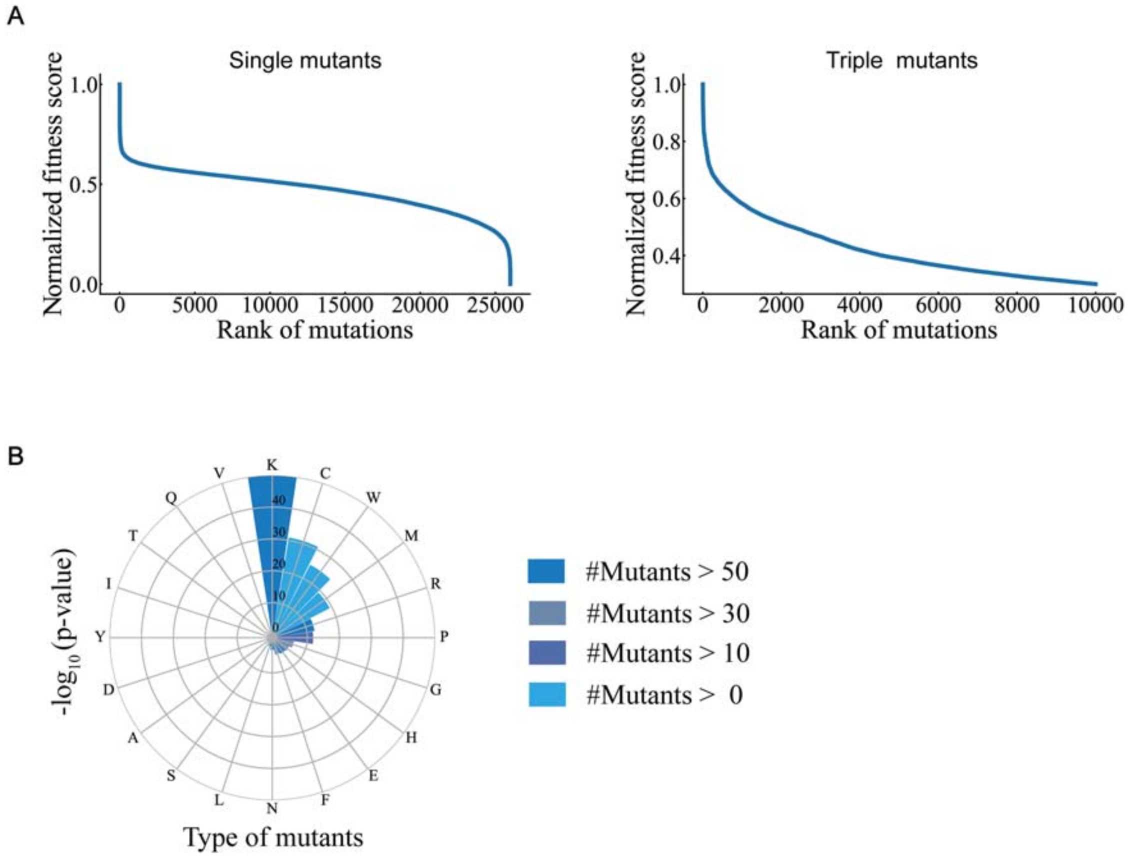

Figure EV1. Enrichment analysis of top-ranked mutants.

(**A**) Normalized fitness score predicted by ProMEP. (**B**) Each polar axis delineates a specific category of mutants (e.g., the K axis denotes X-to-K mutants, while the C axis signifies X-to-C mutants). The radial tick marks correspond to the $-\log_{10}(p \text{ value})$ derived from the enrichment analysis results. The coloration of each bar indicates the count of a particular type of mutant (e.g., among the top 5% mutants, there are 264 X-to-K mutants).

© The Author(s)

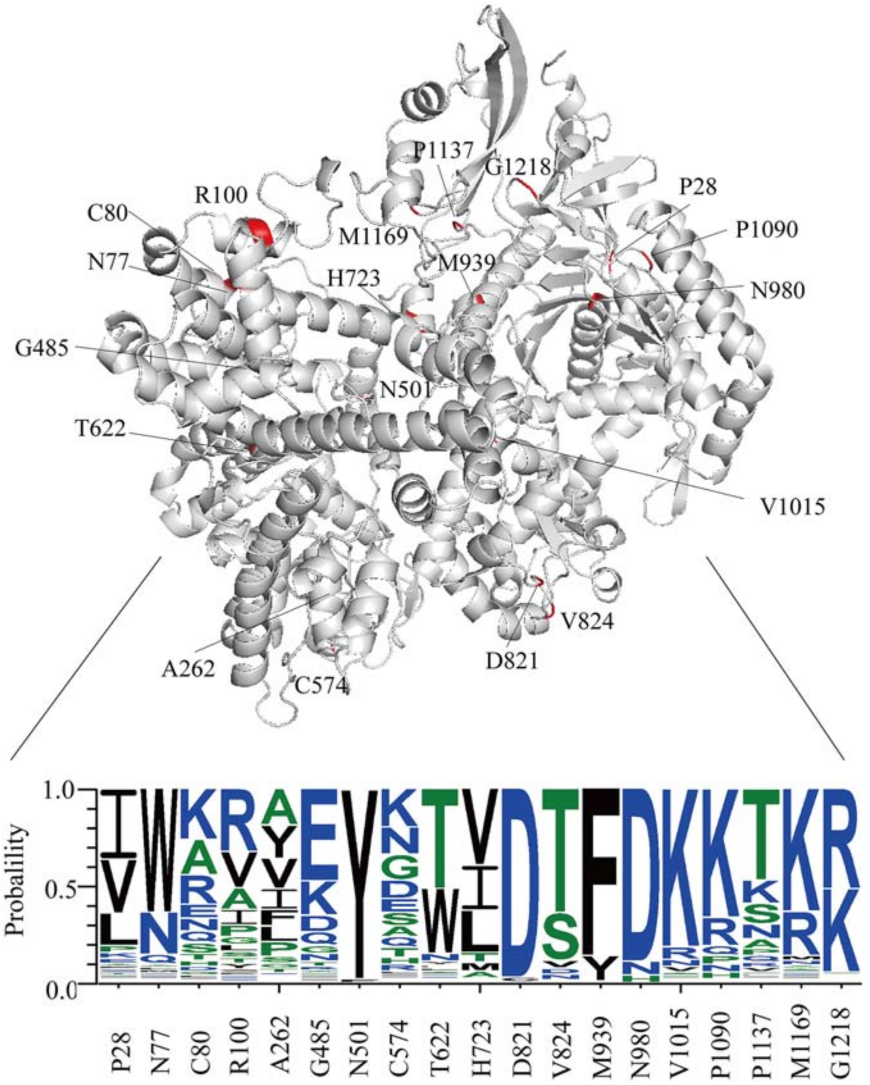

Figure EV2. Probability distribution of Cas9.

The predicted probability distributions of all amino acid types at altered positions within Cas9.

 © The Author(s)

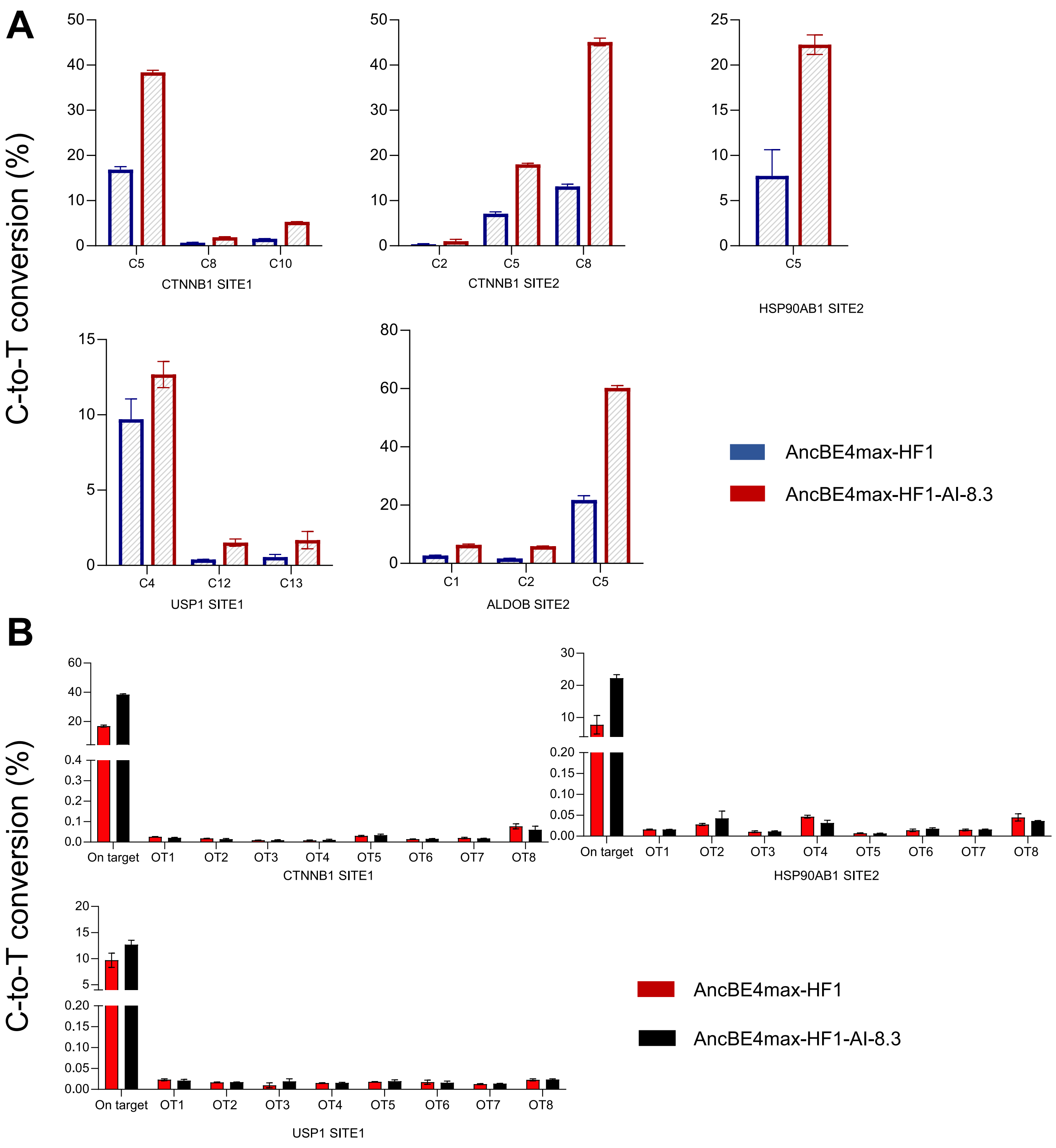

Figure EV3. Editing efficiency and Cas9-dependent off-target effect of HF1-CBEs.

(**A**) The editing efficiency of AncBE4max-HF1 and AncBE4max-HF1-AI-8.3 at all editing positions across five sites in HEK293T cells ($n = 3$ biological replicates). (**B**) Base editing levels for on-target sites (CTNNB1, USP1, and HSP90AB1) and eight corresponding predicted off-target sites were compared between AncBE4max-HF1 wildtype and AI-8.3 groups through NGS analysis ($n = 3$ biological replicates). Data information: data were presented as mean ± s.d.

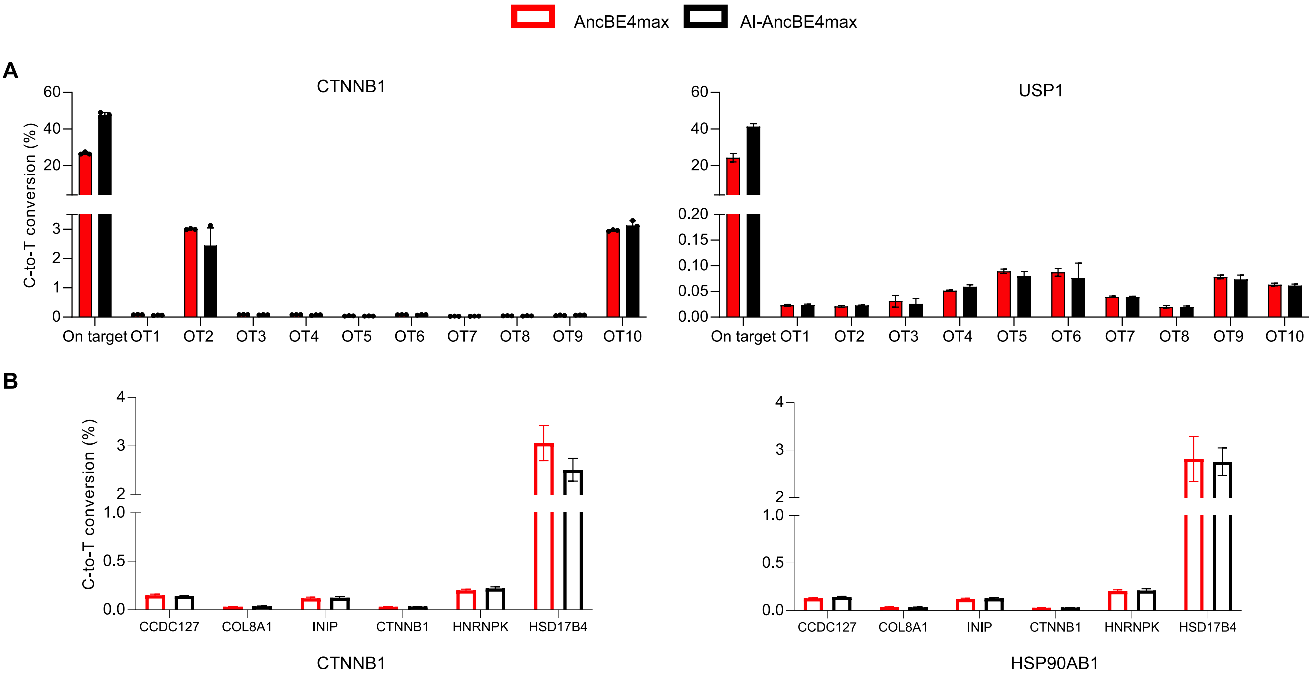

Figure EV4. Off-target analysis of AncBE4max and AI-AncBE4max.

(**A**) Cas9-dependent off-target analysis. Base editing levels for on-target sites (CTNNB1 and USP1) and ten corresponding predicted off-target sites were compared between wildtype and AI-AncBE4max groups through NGS analysis ($n = 3$ biological replicates). (**B**) Cas9-independent off-target analysis. Base editing levels of six dead-enAsCas12f R-loop sites at two Cas9 targets (CTNNB1 and HSP90AB1) ($n = 3$ biological replicates). Data information: data were presented as mean ± s.d.

© The Author(s)
